# In Situ Ptychographic X‑ray Computed Tomography of Fully Hydrated Polyamide Membranes

**DOI:** 10.1021/acs.langmuir.5c04933

**Published:** 2026-01-30

**Authors:** Radosław Górecki, Ronell Sicat, Carla Cristina Polo, Tiago Araujo Kalile, Maria Di Vincenzo, Florian Meneau, Suzana P. Nunes

**Affiliations:** 1 Environmental Science and Engineering Program, Biological and Environmental Science and Engineering, 127355 King Abdullah University of Science and Technology (KAUST), Thuwal 23955-6900, Saudi Arabia; 2 Visualization Core Lab, 127355 King Abdullah University of Science and Technology (KAUST), Thuwal 23955-6900, Saudi Arabia; 3 Brazilian Synchrotron Light Laboratory (LNLS), Brazilian Center for Research in Energy and Materials (CNPEM), Campinas, SP 13083-970, Brazil; 4 Institute of Chemistry, University of Campinas (UNICAMP), Campinas, SP 13083-970, Brazil; 5 Chemistry Program, Physical Science and Engineering, King Abdullah University of Science and Technology (KAUST), Thuwal 23955-6900, Saudi Arabia; 6 Chemical Engineering Program, Physical Science and Engineering, King Abdullah University of Science and Technology (KAUST), Thuwal 23955-6900, Saudi Arabia

## Abstract

Understanding the morphology of membranes under operational conditions is essential for advancing membrane material development for water purification and industrial applications. Traditional characterization methods, such as scanning and transmission electron microscopy, require vacuum environments and sample modifications that could alter membrane structures susceptible to hydration and swelling. Here, we present the first application of synchrotron-based ptychographic X-ray computed tomography (PXCT) for direct in situ nanoscale 3D imaging of hydrated thin-film composite polyamide membranes immersed in water. This method requires no staining, vitrification, or layer separation from the porous support by exposure to organic solvents. It preserves the polyamide membrane’s native state. We studied lab-fabricated membranes containing 2-hydroxy-*N*-(diphenylmethyl) acetamide artificial water channels, revealing significant hydration-induced changes in structure, including up to 51% volume swelling, and increased surface roughness, attributed to the presence of artificial water channels in the polyamide matrix. Morphological features of the ridge-and-valley surface, such as bowl-shaped nodule structures, notably expanded in water. PXCT data were compared to atomic force microscopy measurements, highlighting both the capabilities and limitations of each technique. The in situ PXCT method demonstrated here offers invaluable insight into the hydrated polyamide membrane morphology. The demonstrated high-resolution PXCT method can be applied across disciplines in materials science and life sciences, enabling a detailed analysis of hydrated nanostructures under realistic conditions.

## Graphical abstract

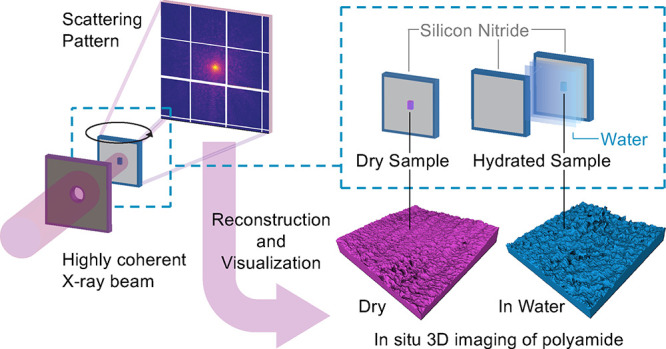

## Introduction

Seawater and brackish water desalination have become essential for life in many countries, currently supplying more than 100 million m^3^ water/day^1^ with a fast-growing demand. A great part (over 70%) of desalination plants operates with reverse osmosis, a membrane-based technology. Their success relies on thin-film composite (TFC) membranes constituted by porous asymmetric supports typically of polysulfone and thin (<300 nm) selective polyamide layers obtained by interfacial polymerization. Modifications are broadly used for nanofiltration in water treatment and other applications. Many approaches have been proposed to tune the performance of TFC membranes for desalination,^2–4^ targeting higher rejection of neutral pollutants, less fouling, higher chemical resistance under cleaning conditions, etc., or to extend their applications to ion separations and recovery and organic solvent nanofiltration.^5–8^ The morphology of most TFC polyamide membranes is typically complex with ridges and valleys, nodules, and inner voids. It results from dynamic processes during the polymerization and is governed by several factors, such as the diffusion rates of reactants to the interface, the porosity of the support layer, the interfacial tension between the organic and aqueous phases, and local temperature fluctuations caused by the exothermic nature of the reaction.^3,9^ A comprehensive understanding of the membrane formation mechanism, the resulting morphology, and the influence of the physical nanostructure of the polyamide thin film on membrane transport and rejection remains a subject of active investigation. This has motivated detailed 2D^10–14^ and 3D^15–24^ imaging analysis by various research groups. There is an intense debate about transport mechanisms through polyamide membranes.^25–29^

Classical methods for membrane morphology investigation include microscopy techniques, such as scanning electron microscopy (SEM) and transmission electron microscopy (TEM). Regular SEM and TEM can provide high-quality 2D data of surfaces, cross sections, and thin specimen sections with nanometer resolution. Free volume or pore sizes in the subnanometer range are hardly accessible with microscopy but can be indirectly estimated by rejection tests of solutes of different sizes or by positron annihilation lifetime spectroscopy (PALS), which is not an imaging method.^12,30^ AFM depicts the membrane surface with a 3D visualization but does not supply subsurface information. Detailed 3D images of the internal membrane pore structure can be obtained by applying advanced electron microscopy methods, such as focused ion beam (FIB) SEM, serial-block face (SBF) SEM, and transmission electron tomography.^23,31,32^ These imaging methods offer resolutions of a few nanometers (SBF and FIB) or around 1 nm in the case of TEM tomography. The sampling volume varies with the methods. While TEM has the best resolution, it allows the lowest sampling volume. Typically, the samples for TEM tomography of polymer films are 100–300 nm thick and the lateral field of view (*X* and *Y*) is about 100–500 nm when working with high resolution, giving a sampling volume of about 0.075 μm^3^. Besides the restricted sampling volume, although electron microscopy has excellent resolution, there are other limitations. Electron microscopy analyses require the specimen to be imaged under high vacuum, membranes are regularly analyzed in the dry state, and often, the analysis involves multiple preparation steps, such as staining with heavy metals, embedding in resins, and dissolution of the porous substrate to isolate the membrane selective layer.^14,15,17,19,23,31^ These steps are essential but might add artifacts to the microscopy analysis. It is challenging to apply conventional electron microscopy methods to analyze the TFC polyamide active layer under conditions that represent the operational environment, starting from observations in water. The polyamide active layer swells significantly in the hydrated state. Changes in the layer thickness and weight variation when exposed to water have been precisely quantified using ellipsometry and quartz crystal microbalance.^13^ Neutron scattering enables the investigation of the water dynamics in polyamide membranes.^12,33^ These are all accurate but nonimaging methods. AFM enables imaging of the membrane surface in water, indicating how the roughness is affected by swelling.^34^ However, until recently, no 3D image of the full polyamide layer under water was available, leaving open hypotheses based on speculations of how performance and morphology are correlated.

The advance of cryo-TEM enabled the elegant tomographic image and analysis of hydrated polyamide membranes reported for the first time by Yao et al.,^35^ followed by Li et al.^36^ They studied the hydrated morphology of the isolated active layer of commercial seawater and brackish water membranes, having resolutions in the range of 3–8 nm. They confirmed changes of film thickness and surface roughness and quantified the accessible surface area of the rough polyamide membrane for water transport.^35,36^ The TFC membrane is multilayered. In the cryo-TEM investigation of Yao et al.,^35^ the polysulfone membrane support was dissolved in dichloromethane and the isolated 150–200 nm thick polyamide layers were hydrated by exposure to water vapor instead of liquid water to avoid excessive swelling, high sample thickness, and formation of vitrified ice. The membrane surface was then wetted with a minor amount of liquid water. The hydrated membrane was plunge frozen in liquid ethane and imaged in cryo-TEM. Typically, for TEM tomography, a series of images are acquired at tilting angles from −60 to 60° and used to obtain a 3D-reconstructed image. Li et al.^36^ had slightly different preparation steps. The polysulfone support was dissolved in *N,N*-dimethylformamide. The isolated polyamide layer was immersed in a gold aqueous solution to increase the contrast. The layer was removed and dried, and 4 μL of the same solution was deposited on the membrane surface before plunge freezing in liquid ethane.

All of the methods mentioned above provide complementary insights and contribute to a comprehensive understanding of the TFC membrane morphology and transport characteristics. However, none has yet enabled 3D imaging of membranes directly in liquid water or under operational conditions without detaching from the support, staining, or vitrification. This is now possible through ptychographic X-ray computed tomography (PXCT) using synchrotron radiation. PXCT imaging operates without vacuum and enables nanoscale 3D analysis of soft-matter samples in their native state without the need for modifications.^37–39^ Until recently, X-ray computed tomography of soft matter had the advantage of providing images with larger sampling volumes than electron microscopy but lacked the resolution necessary to resolve nanostructured membranes. Advances have recently enabled frontier experiments resolving the morphology of block copolymer samples with sub-15 nm features using PXCT.^40,41^ For the analysis, one of the phases was etched out by UV exposure and extraction with ethanol, and the remaining voids were filled with gold, which provided an excellent contrast for the analysis of the dry samples. The analysis of unstained polymeric samples like TFC membranes containing only carbon, nitrogen, and oxygen is even more challenging.

We recently reported the first 3D visualization of dry polymeric nanoporous hollow fiber membranes performed by PXCT at 26 nm resolution over a large field of view.^38^ Here, we apply PXCT to TFC membranes. More specifically, the membranes investigated in this work are polyamide-based, prepared by interfacial polymerization with integrated assemblies of 2-hydroxy-*N*-(diphenylmethyl) acetamide (HNDPA), which form channels preferentially permeable for water. This membrane was recently reported by our group and outperforms commercial water desalination membranes in terms of water flux, salt, urea, and boron rejection.^42^ Particularly relevant is the investigation of the membrane morphology in the hydrated state.

To the best of our knowledge, this is the first report of in situ synchrotron imaging of unmodified soft-matter samples in water. The in situ PXCT method presented here offers unique access to the nanoscale structure of membranes close to their operational state and can be extended to other porous systems across material and life sciences.

## Results and Discussion

### PXCT Preparation Steps

PXCT analysis does not require vacuum, a conductive surface, ultrathin slices, or freezing. Only the nonwoven polyester backing was mechanically detached from the membrane. The polyamide layer remained attached to the polysulfone asymmetric porous support, eliminating any dissolution step in the organic solvents. Blocks of a 15 μm × 15 μm membrane system (polyamide on polysulfone support) were cut by laser microdissectioning from the top down through the entire porous support structure. This enables imaging of a large volume of samples with detailed investigation of different parts of the membrane, from the dense selective polyamide layer to the asymmetric support porous structure and the interface between them. This is relevant since the intrusion of the polyamide layer during polymerization is a factor that, in many cases, increases the transport resistance or makes the interpretation of results more complex. The block samples were placed on a silicon nitride membrane for PXCT analysis. For the observation of hydrated samples, they were placed between two silicon nitride membranes separated by a frame. Water filled the space between the Si_3_N_4_ membranes (Figure 1), and the membrane sample was allowed to hydrate for 1 h prior to PXCT measurements, mounted onto the holder, and transferred to the synchrotron beamline experimental hutch. To avoid evaporation during the measurements, the silicon nitride frames were glued together with UV-curing glue. In this way, the membrane could be directly observed in water without any potential alteration by freezing or additional preparation steps.

**Figure 1:**
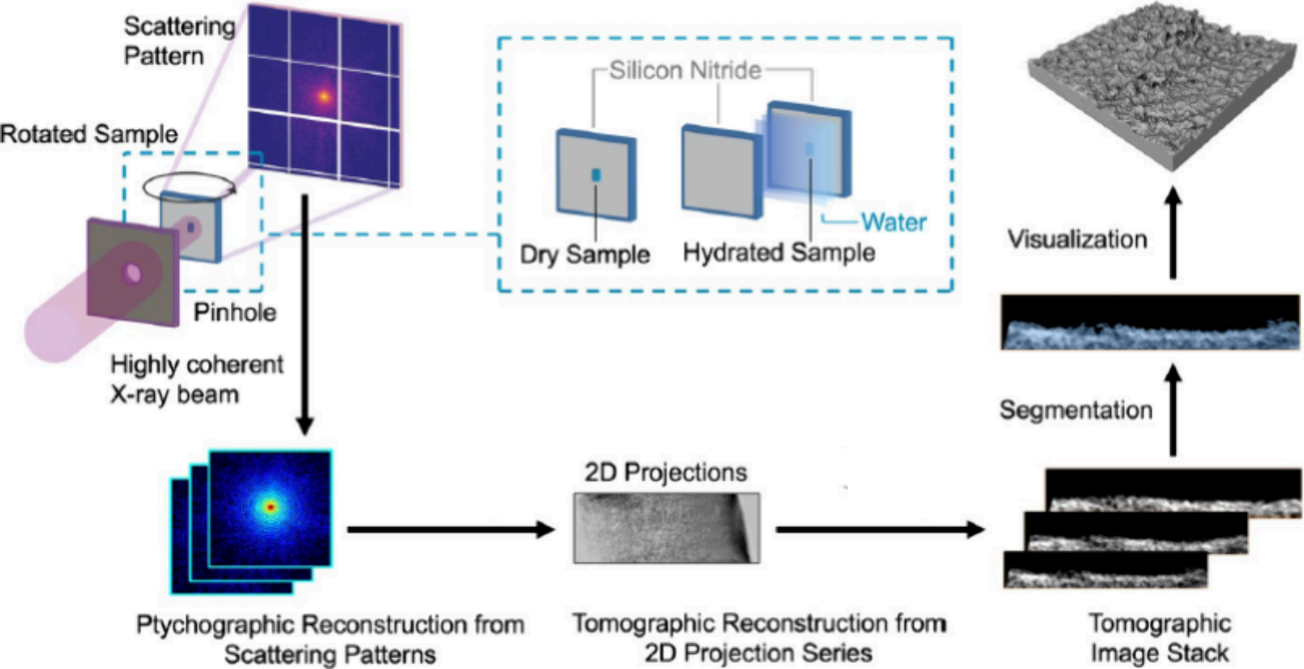
Workflow for ptychographic X-ray computed tomography (PXCT) in situ, including measurements and data processing.

### Ptychographic X-ray Computed Tomography Data Collection

The three-dimensional nanoscale morphology of both dry and hydrated polymer membranes was analyzed by ptychographic X-ray computed tomography at the Cateretê beamline^43^ of the SIRIUS fourth-generation synchrotron facility.^44^ The measurements were conducted using a coherent X-ray beam energy of 6 keV, selected by a 4-bounce crystal monochromator from the undulator source illumination. For the dry sample, a 10 μm pinhole aperture was employed, while a 15 μm aperture was used for the hydrated specimen to define the confined coherent illumination on the sample. The samples were raster-scanned, and coherent diffraction patterns were recorded downstream with a PIMEGA 540D detector positioned 15 m from the sample, providing high-resolution data acquisition at a pixel size of 31 nm. Scanning was performed along a linear XY trajectory, yielding 648 and 315 individual diffraction patterns for dry and hydrated samples, respectively. A constant exposure time of 150 ms per point ensured an adequate signal while limiting radiation-induced damage. The scanning grid incorporated an average 2 μm step size with an 80% overlap, and random perturbations were introduced to each step (±50%) to suppress periodic reconstruction artifacts.^45^ The tomographic datasets comprised 469 projections for the dry membrane and 650 projections for the hydrated one, with respective angular coverages of 126 and 116°, resulting in missing wedges of 54 and 64°, as depicted in Figure S1. The angular coverage was limited due to the use of a silicon nitride frame, which at high angles obstructs the X-ray beam. Ensuring that the sample holder remained sealed and the membrane immersed in water during the whole PXCT experiment was essential; thus, in the case of bubble formation or observed water evaporation inside the two silicon nitride windows, the PXCT experiment was either stopped or repeated with a new hydrated sample.

### X-ray Data Processing and Image Reconstruction

The acquired coherent X-ray diffraction pattern datasets were processed using the PtychoShelves software package^46^ using the graphical processing units (GPUs) of KAUST Ibex Supercomputer, enabling a high-throughput processing of such large datasets. To fully reconstruct images from the diffraction patterns, information on both the wave amplitude and phase is needed. The amplitude is directly proportional to the square root of the intensity, which is measured by photo counts of signals reaching the experiment detector. The phase must be retrieved using iterative algorithms. Regions of 1800 pixels × 1800 pixels of each of the diffraction patterns were used for phase retrieval. As an iterative algorithm, we applied the difference map, which is based on differences of pairs of elementary projections to guide the reconstruction.^47,48^ The maximum likelihood^49^ was used, with 1000 iterations and position corrections^50^ were initiated after 200 iterations of ML.

The resulting series of 2D projections covered a field of view of 70 × 33 μm^2^ for the dry and 68 × 16 μm^2^ for the hydrated sample. The projection series of both samples were further processed with PtychoShelves and aligned according to Guizar-Sicairos et al.^51^ and Odstrči et al.,^51,52^ using cross-correlation alignment, mass fluctuation-based vertical alignment, and tomographic consistency alignment.^53,54^ Final tomographic reconstructions were obtained by using a filtered-back projection algorithm with a Ram-Lak filter. The aligned sinograms after the tomographic consistency alignment obtained from PtychoShelves tomographic reconstruction script for dry and hydrated samples are shown in Figure S2. The projections for dry and hydrated samples before and after alignment are presented in the Supporting Information in Videos S1 and S2 for the dry sample and in Videos S3 and S4 for the wet sample. They are the collection of images from all angles, reconstructed from the scattering patterns. These are raw images before any segmentation. The tomographic reconstruction of the dry sample was 2240 × 2240 × 1088 voxels, and the tomographic reconstruction of the hydrated sample was 2208 × 2208 × 512 voxels. The final projection voxel size was 31 nm × 31 nm × 31 nm for both datasets. The resolution, estimated by localized line-scan analysis at the interface of the active layer and the background, was 70 ± 5 and 70 ± 3 nm for dry and 69 ± 5 and 69 ± 2 nm for the hydrated membrane in the *XY* and *YZ* planes, respectively. The resolution was estimated from the full width at half-maximum (fwhm) of the intensity profile taken across a sharp interface in the reconstructed image. The fwhm reflects how broad the transition appears between a dark and a bright region, providing a direct estimate of the smallest resolvable feature size in the unsegmented dataset.^55–57^ Figure 2 and Videos S5 and S6 show a 3D reconstruction of the presegmented data obtained from the collection of images in Videos S2 and S4, where the porosity and the selective layer are more evident. Examples of 2D presegmentation membrane images are shown in Figures S3 and S4.

**Figure 2:**
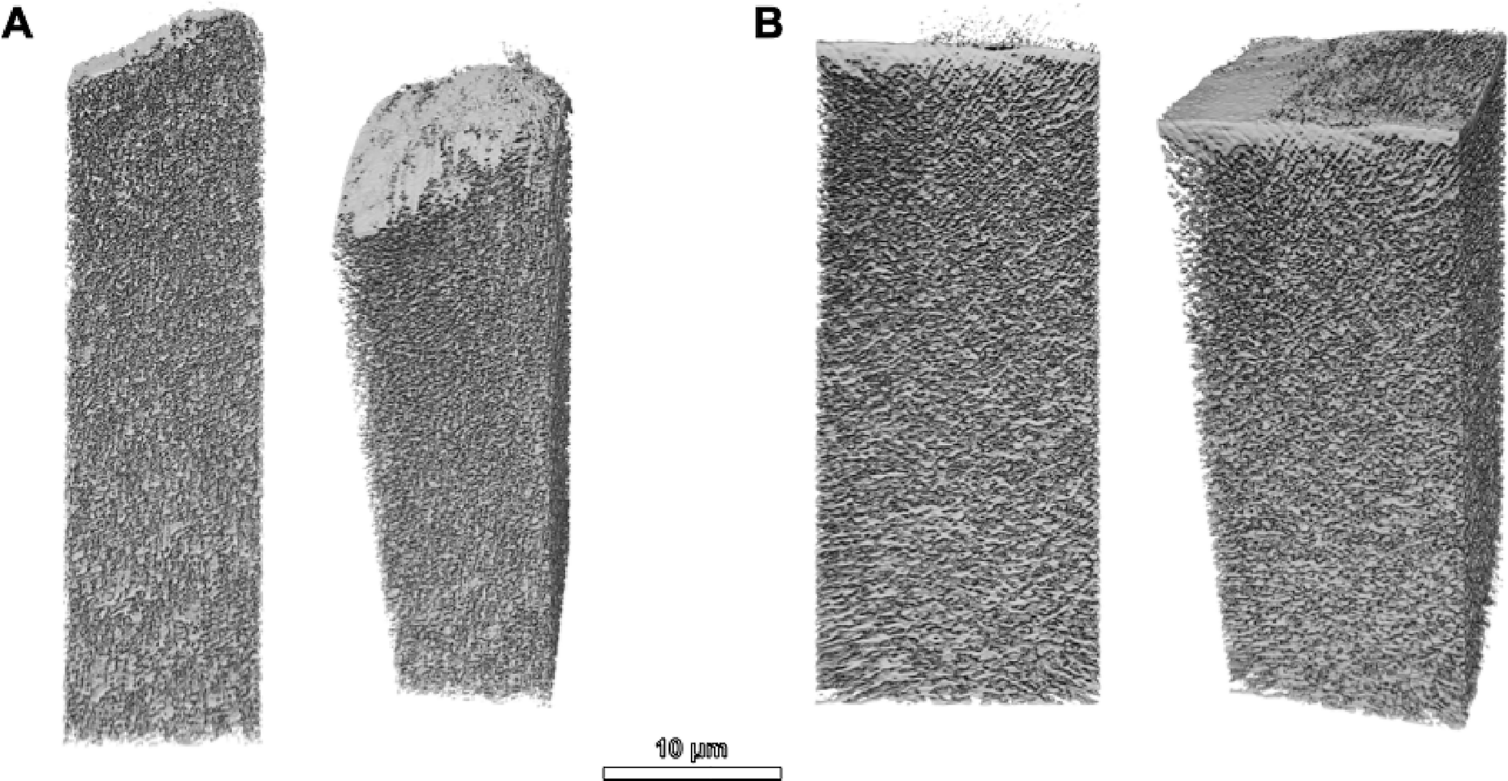
3D visualizations of the presegmented data of the polysulfone support with the polyamide active layer, prepared by applying the unsharp masking module and iterative median filter in Avizo for (A) dry membrane and (B) hydrated membranes. The background noise is present on the top polyamide surface and particularly pronounced for the hydrated membrane sample.

### 3D Visualization of Dry and Hydrated Morphologies of Polyamide Membranes

The 3D visualization of the reconstructed PXCT volumes was carried out with Avizo 3D. From the reconstructed 3D volume of the whole scanned region of the membrane, regions of interest were selected for detailed processing. The regions of interest correspond to 5 μm × 5 μm surface areas of the membrane. The regions of interest correspond to 6 μm × 6 μm surface areas of membrane. We excluded regions affected by missing wedges, where the image acquisition could have been compromised by incomplete angular coverage. For the detailed visualization of the polyamide layer, the next step was the manual segmentation on a slice-by-slice basis since the automated segmentations provided by the Avizo 3D software were not completely satisfactory. Examples of orthogonal slices before and after the manual segmentation are presented in Figure S5. For the 3D visualization, we used volume rendering with cubic interpolation to enhance the image quality and reduce aliasing artifacts^58^ (the comparison of visualizations without and with cubic interpolation is provided in Figure S6). While all quantitative data regarding roughness and volumes provided in this study are based on the unmodified 31 nm × 31 nm × 31 nm voxel datasets, cubic interpolation provided enhanced visualizations, and we used these images to estimate lengths of visualized structures more accurately. Final 3D visualizations of dry and hydrated membranes are presented in detail in Videos S7 and S8, respectively.

### Morphologies of the Dry and Hydrated TFC Layers

The detailed visualizations of dry and hydrated polyamide layers revealed clear differences in their morphologies. First, 2D surface images are shown in Figure 3. The morphology of the dry polyamide that consists of characteristic ridge-and-valley structures, with round and elongated nodular structures, is typically seen by SEM, as in Figure 3A. Nodules with ridges imaged by SEM have a thickness ranging between 20 and 60 nm. Similar morphological features were visualized by PXCT in Figure 3B. The thickness of the observable nodular ridges imaged by PXCT ranged from 31 to 80 nm in the dry state (Figure S7). However, the nodules are slightly different from those imaged by SEM. They are not collapsed, which might indicate that vacuum and preparation conditions affect the observed morphology of dry membranes. The 2D images of the dry and hydrated layers obtained by PXCT were visibly different with the hydrated nodules being clearly more inflated (Figure 3C and Figure S8).

**Figure 3:**
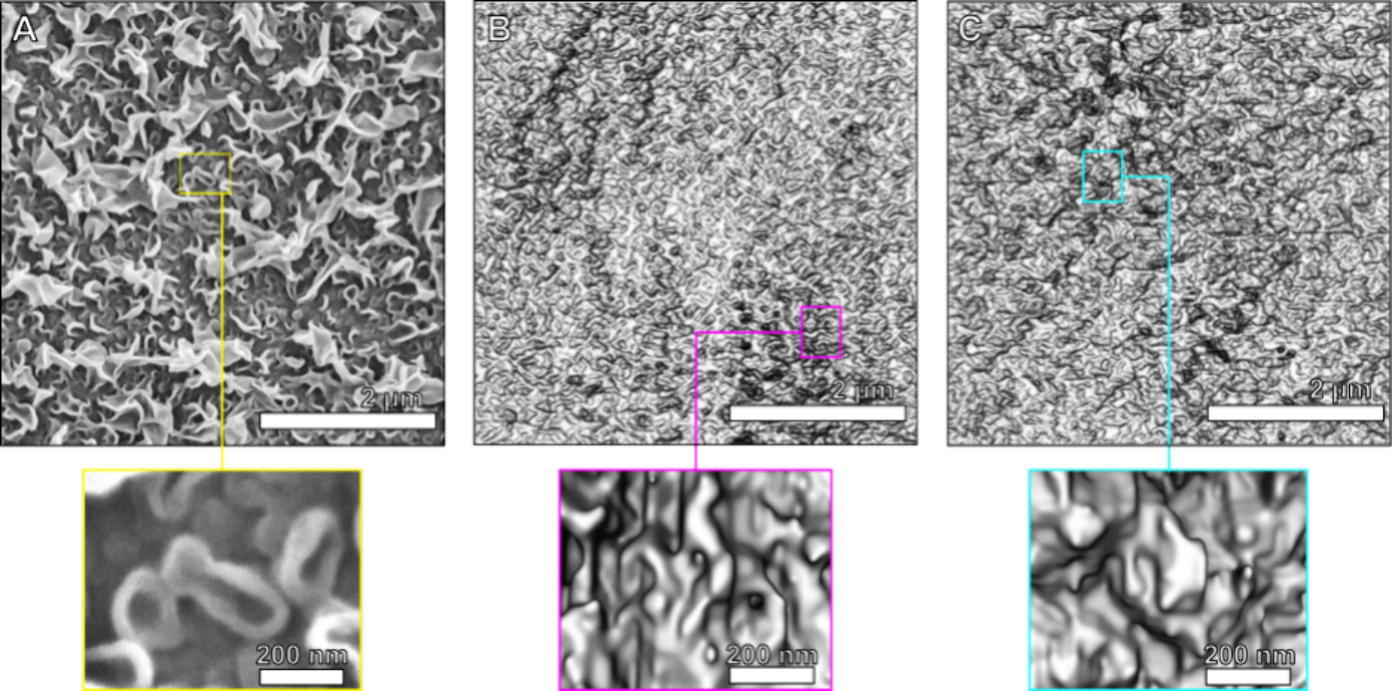
2D images of the membrane surfaces. (A) SEM image. PXCT visualization of (B) a dry and (C) a hydrated membranes. Close-up images showing bowl-like nodules and the different features observable at the dry and hydrated states.

The differences between the dry and hydrated states become even clearer when analyzed in detail in PXCT 3D visualization; the results of PXCT are presented in Figure 4A,B. To provide detailed insight and understanding of the polyamide swelling in the hydrated state using visualized results from PXCT, we selected similar features in the visualizations of both states (Figure 4C and Figures S8 and S9) and estimated the average volume changes of various morphological features of the polyamide. The volume in the swollen state was 30–51% higher (the specific volumes are listed in Table S1). These values are higher than those recently published for commercial brackish water and seawater membranes, estimated with the use of cryo-electron tomography, which were equal to 32 and 7%, respectively.^35,36^ We analyze local features of a similar form. Features that protrude more prominently from the surface already in the dry state appear bulkier in the hydrated state in comparison to those more integrated into the surface and less elevated. This aligns well with the molecular simulation studies of Zhang et al.,^59^ which suggest a higher degree of swelling for thinner polyamide layers. More protruding features are more exposed to water. According to Li et al.^36^ recently published cryo-electron tomography study, the primary effect of the membrane swelling is the expansion of the hollow nodular structures, which are also undoubtedly present in our membrane, as proven by SEM imaging (Figure S7). The internal hollow parts of the nodules are usually in the size range of 20 nm, thus being close to and below the current PXCT technique’s resolution limits. Therefore, internal details of the nodules could not be revealed by PXCT. On the surface of polyamide, besides typical bowl-like nodules, we observed larger, more elongated, and deformed structures, which are frequently observed on the active layer of polyamide TFC membranes, particularly at the surfaces of brackish water membranes, having heights of about 200 nm in the dry state (Figure 4D,E). Their hydration is very pronounced as they change dramatically in a hydrated state, elongating and reaching heights of 400 nm (Figure 4D).

**Figure 4:**
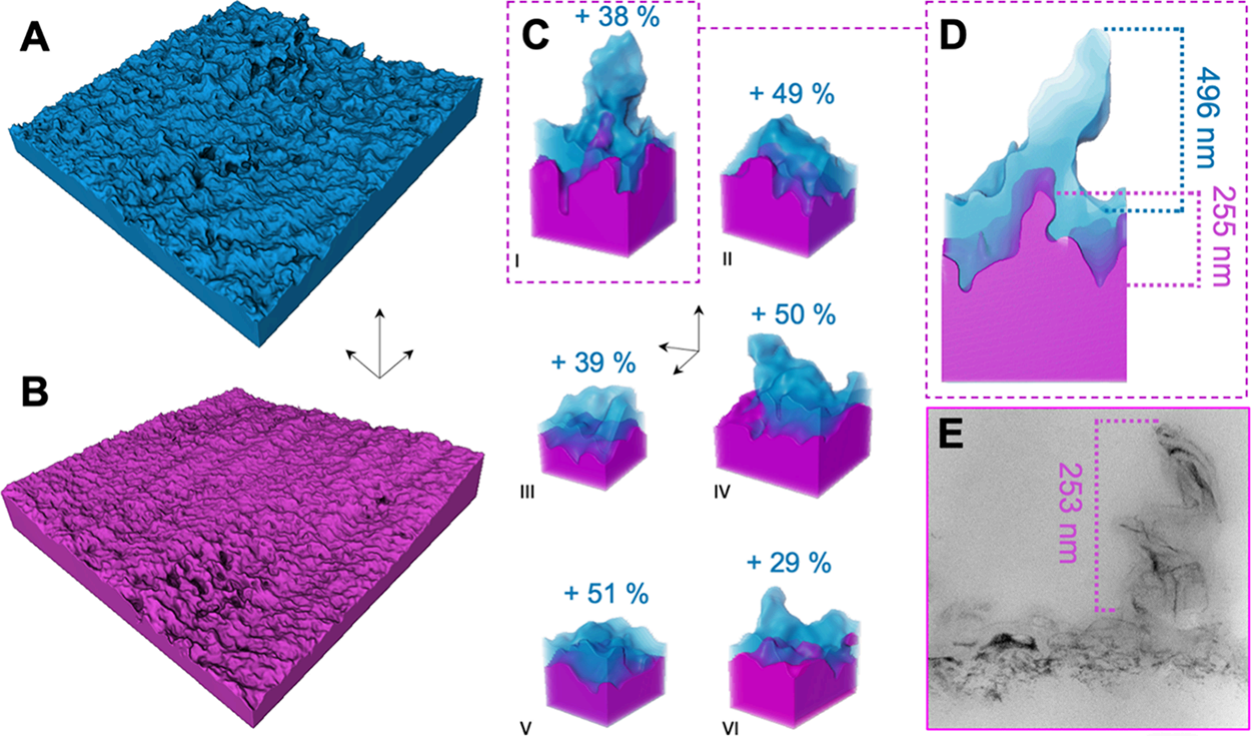
Detailed visualization of dry and hydrated membranes. (A) PXCT 3D visualization of the hydrated membrane, 5 × 5 μm area. (B) PXCT 3D visualization of the dry membrane, 5 × 5 μm area. Scale bars for panels (A) and (B) are 500 nm in all directions. (C) Comparison of the PXCT visualizations of the structures of a similar morphology at dry (magenta) and hydrated (blue) states. Figures obtained by overlapping images of panels (A) and (B), marking the dry image contribution as magenta, and the remaining part of the wet membrane image as blue. Scale bars in panel (C) are 200 nm in all directions. The roman numerals (I–VI) refer to the table volumes presented in Table S1. (D) Comparison of the cross sections of wet and dry elongated features of polyamide. (E) Similar features of polyamide observed in the ultrathin (70 nm) section by transmission electron microscopy, stained with osmium tetroxide (dry state), dense black parts of the polyamide are clusters of 2-hydroxy-*N*-(diphenylmethyl) acetamide nanochannels. Transmission electron micrograph is reproduced with permission from Di Vincenzo et al., 2025.^42^ Copyright 2025 Elsevier B.V.

There are multiple reasons why we observed higher swelling than previously reported. First, the membranes investigated here have HNDPA self-assemblies integrated into the polyamide layer. However, as we reported before,^42^ the HNDPA membrane NaCl rejection rates are even higher than those of the commercial SW30 membrane; thus, an increased swelling cannot be associated with a reduction of cross-linking density. The HNDPA channels and their hydrophilic domains are densely dispersed in the polyamide (Figure 4E) and are highly accessible to water. They allow water to be transported into a deeper, confined layer of the polyamide.^42^ Because these supramolecular assemblies are homogeneously distributed throughout the film rather than clustered at the surface, water uptake occurs throughout the full depth of the polyamide layer, leading to a more uniform and pronounced volumetric swelling compared to that of membranes where hydrophilic moieties are localized only near the surface. The small features observed for HNDPA membranes by SEM and TEM, assigned to the HNDPA assemblies, are out of the current resolution limits of PXCT and could not be distinguished. Besides the presence of HNDPA, there might be more significant reasons for the observed swelling. In the work presented here, the membranes were not subjected to compaction prior to measurement; thus, the nodular empty space can be higher than in the previous studies. In our work, the polyamide thin films were analyzed without separation from the polysulfone support, thereby avoiding exposure to organic solvents, such as dichloromethane or chloroform. This step prevents potential solvent interactions with the polymer, particularly with less-cross-linked regions of the polyamide, that could result in solvation of less- and non-cross-linked parts of the polyamide, altering the native morphology formed through interfacial polymerization. Additionally, and more importantly, by using PXCT, all measurements were conducted under ambient conditions rather than under vacuum, minimizing structural changes that vacuum exposure might induce, such as the collapse of the nodules, especially in the dry state. We could study the sample directly immersed in water, and as reported previously, the liquid water uptake in polyamide membranes is higher than the water vapor uptake,^13,35^ resulting in a larger water fraction being confined within the polyamide,^33^ of which a large part will be bulk, free water.^12^ Direct measurements in water allowed us to omit the vitrification step, which could induce water removal by blotting.

Figure 5 compares the complementary results of AFM and PXCT for both dry and hydrated membranes. AFM analysis gives information on the membrane surface roughness and can be conducted in water as well. However, it is a surface analysis method, no information is possible on the sublayer porosity, and the depth of analysis depends on the thickness of the AFM probe and its ability to access the narrow valleys of the surface’s topographic relief. We used the PXCT visualizations to generate height maps (Figure 5A–D), extracted the height data, and compared it to the information obtained from AFM measurements of the membrane surfaces in dry and hydrated states. This data are summarized in Table S2. The arithmetic average roughness (*R*_a_) and root-mean-square roughness (*R*_q_) of dry and hydrated polyamide layers based on PXCT were lower than the values calculated from AFM measurements. The *R*_a_ values for the dry layers were equal to 40 and 93 nm for PXCT and AFM, respectively, while *R*_q_ values were 50 nm (PXCT) and 111 nm (AFM). For hydrated samples, the roughness increased. *R*_a_ values were 92 nm (PXCT) and 120 nm (AFM), and *R*_q_ values were equal to 109 nm for PXCT analysis and 150 nm for AFM-based calculations. Polyamide swells upon hydration due to the uptake of water molecules, which interact with hydrophilic functional groups such as amide and carboxylic acid moieties in the polymer matrix. These interactions lead to hydrogen bonding between the water and polymer chains, causing the polymer network to expand and increase in volume. Additionally, the infiltration of water into existing nanoscale voids further contributes to the swelling effect, water-filled nodules balloon and rise, resulting in greater surface roughness when the membrane is hydrated.^13,35,36^ To further understand the effect of the technique used on the quality of the measurements, we calculated the skewness (*R*_sk_) and kurtosis (*R*_ku_) of the surfaces. The *R*_sk_ of dry samples based on PXCT was equal to −0.09, suggesting more valleys on the surface, while based on AFM, it was equal to 0.16, suggesting more peaks on the surface. The discrepancy in the values provided by the two techniques could be due to a difference in the resolution. The lateral AFM resolution with a fine probe is higher than the current PXCT resolution. The contribution of a larger density of smaller structures on the surface would lead to an apparent higher roughness value. On the other hand, the AFM probe might not be able to penetrate deeper structures of the rough surface of polyamide that are obstructed from the top. The inaccessible cavities in the structure that cannot be probed by AFM are presented in detail in Figure 5E,F. The *R*_sk_ values for hydrated samples were positive for both AFM and PXCT methods and were 0.23 and 0.02, respectively, suggesting more peaks than valleys in the measured samples, though again, the sensitivity of AFM to study structures deeper in the complex surface elevates the *R*_sk_ (Figure 5E–H). The *R*_ku_ values based on PXCT were 3.31 for the dry layer and 2.57 for the wet layer. Thus, suggesting the structures changing from sharp-edged to more rounded profiles with hydration, as filling of the nodular voids and swelling of the layer results in a ballooning effect, which is well aligned with previously reported cryogenic transmission electron tomography imaging.^36^ The AFM-based calculations, however, suggest contradictory, as *R*_ku_ values equal to 2.81 for dry and 3.80 for wet samples, suggesting that rounder profiles change to sharper shapes. These readings are directly impacted by the structures becoming bigger in a swollen, hydrated state. The details of such larger structures are easier to resolve with the use of the PXCT technique, while studying the extended hydrated features becomes more difficult with AFM: First, because the structure of the soft material becomes more difficult to resolve in water, as the force sensitivity in water is greatly reduced.^60,61^ Second, when the larger differences in height arise, the error in measurements of the exact feature shape increases due to the finite geometry of the AFM tip.^35,62^ This also explains the differences in calculations for the surface area based on PXCT and AFM. For the dry samples, the surface area of a region of interest of 2 μm × 2 μm was equal to 5.5 μm2 for PXCT, as well as 8.8 μm^2^ for AFM. For the hydrated samples, the surface areas were higher and for PXCT equaled 10.6 μm^2^, and 9.2 μm^2^ for AFM. Here again, PXCT becomes advantageous for resolving large swollen structures, as the detailed morphology underneath the top surface of swollen structures is unveiled and considered for the calculation of the surface area, resulting in a higher value than using the AFM reading. The resolution limitations of PXCT, however, miss detailed, smallest features in the dry sample, which results in lower surface area values for PXCT in comparison to AFM. The orthogonal slices of hydrated and dry samples that clearly represent a change in visible features are presented in Figure 5G,H.

**Figure 5:**
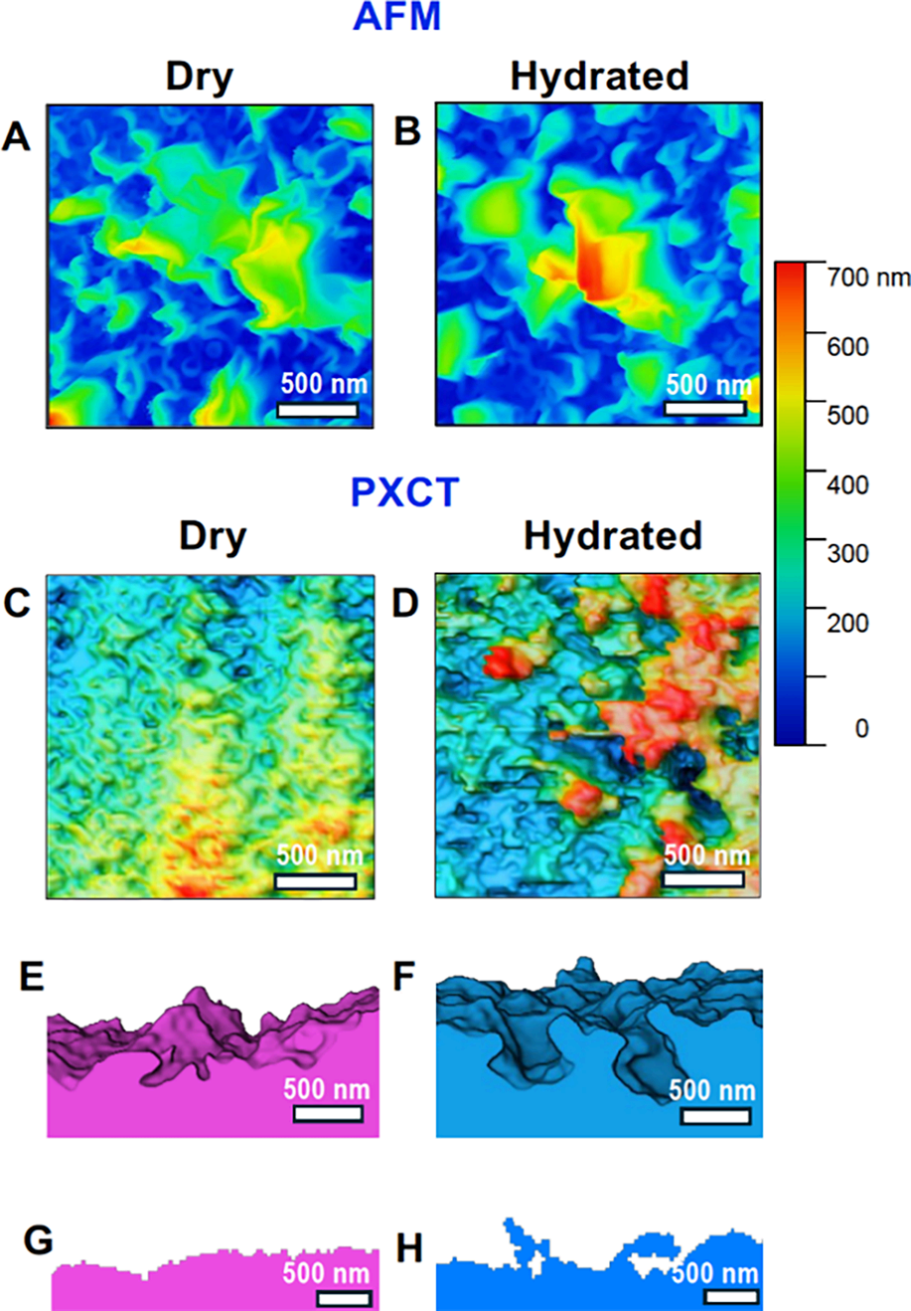
(A, B) AFM and (C, D) PXCT height maps of the surface of (A, C) dry and (B, D) hydrated polyamide layers. Additional information was provided by PXCT: orthogonal slices of the 3D visualization of (E) dry and (F) hydrated polyamide layers; orthogonal slices of (G) dry and (H) hydrated polyamide layers, illustrating the level of morphological details.

Surprisingly, according to our observations, the surface area and the surface roughness parameters increase upon hydration in contrast to what has been recently reported by Yao et al.^35^ Their cryo-TEM tomography results indicate that the surface roughness of brackish water and seawater polyamide membranes did not change significantly upon hydration in comparison to the dry state, despite their significant increases in volume and thickness. The authors attribute such observations to the change in the ridge-and-valley structures after hydration, expanding and flattening of ridges, and valleys becoming smaller, resulting in a flatter membrane surface.^35^ According to our observations that are based on both AFM and PXCT measurements of water-immersed samples, the swelling of round nodules and elongated more deformed structures does not result in flattening, but due to extension in both lateral and vertical directions, the height differences increase, leading to larger roughness parameters. Therefore, increased roughness, as well as increased swelling, can be attributed to the effect of HNDPA channels distributing water also within the confined layers of the polyamide and the fact that the polyamide layer has not been separated from the support. The even dispersion of HNDPA assemblies ensures that water is taken up not only in the uppermost nodules but also within subsurface regions, amplifying the vertical expansion and contributing to the overall increase in roughness measured by both PXCT and AFM. Increased water distribution in the bottom part of the polyamide and swelling of the hollow nodules present at the interface between the polyamide layer and the polysulfone support result in lifting the structures on the polyamide–water interface upward, thus increasing the roughness. The HNDPA assemblies are rich in hydroxy groups. With a homogeneous distribution and incorporation into the polyamide layer, the hydrophilicity of the selective layer is increased. This directly reduces the Gibbs energy of mixing between the polymer matrix and the solvent by reducing the enthalpic contribution. As described by a classic thermodynamic models describing the polymer network swelling in solvents,^35,63,64^ the total Gibbs energy is a sum of the regular Gibbs energy of mixing plus an additional entropic contribution linked to the stretching of the network during swelling. Higher hydrophilicity enthalpically promotes swelling. The entropic contribution to swelling is related to the degree of cross-linking. Tightly cross-linked polyamide has shorter segments between the cross-linking points, which restricts the change in entropy, increases the Gibbs energy, and reduces swelling. The degree of cross-linking of the polyamide layer directly affects the salt rejection and the water permeance of a TFC membrane, with less cross-linked membranes tending to have lower rejection and higher permeance. While HNDPA membranes have high water permeance, their salt rejection is high; we assume that the water transport is preferentially favored through points rich in HNDPA channel assemblies, while the cross-linking densities of polyamide segments remain comparable to those of classical reverse osmosis seawater desalination membranes. Therefore, similar entropic contributions to the swelling Gibbs energy are expected.

## Conclusions

We demonstrate the power of high-resolution PXCT to directly visualize the 3D nanoscale structure of soft polymeric materials immersed in water with a focus on polyamide TFC membranes. It provides unique and valuable morphological information under conditions that are inaccessible to other technologies. PXCT enables large volume visualization, revealing key structural features of the membrane, including the polyamide layer, the porous polysulfone support, and their interface. Unlike conventional electron microscopy and cryo-TEM, PXCT allows in situ imaging directly in water, eliminating steps of film isolation, vitrification, and staining. Upon hydration, the PXCT imaging revealed significant morphological changes in the polyamide with supramolecular assemblies of HNDPA. These changes included volume expansion, increased surface roughness, and a larger surface area in the hydrated membrane compared to the dry sample. Higher swelling was observed compared with recent cryo-TEM studies of desalination and brackish water membranes. This difference is attributed to both the unique composition of our developed membranes, with incorporated hydrophilic water channels, which facilitate the water uptake deeper into the polymer matrix, and second to the absence of sample preparation artifacts, as PXCT allows direct observation in water. In summary, PXCT offers unprecedented access to hydrated nanostructure observation of polymeric membranes with the potential of broad applicability across materials and life sciences.

## Materials and Experimental Methods

### Membrane Materials

Commercial flat sheet polysulfone ultrafiltration membranes (Solecta, M-PS20-GPP) were used as a support layer to fabricate reverse osmosis membranes. All solvents were HPLC grade. Ethanol and hexane were purchased from VWR International Ltd. Trimesoyl chloride (TMC) 98%, m-phenylediamine flakes 99%, sodium chloride (NaCl) ≥99.5%, urea powder >99%, and sodium metabisulfite (Na_2_S_2_O_5_) ≥98% were purchased from Sigma-Aldrich. Chemicals were dissolved in deionized (DI) water or hexane prior to membrane fabrication. DI water was obtained from a Milli-Q purification system (MilliporeSigma, MA, US). All of the reagents and solvents were used without any further purification or anhydrization. Commercial SW30HRLE (DuPont, FilmTec) membranes were used as received for a comparative study.

### TFC Membrane Fabrication

HNDPA artificial water channels were synthesized by a straightforward one-step reaction as reported in detail in our previous work.^42^ Selective polyamide hybrid layers incorporating HNDPA artificial water channels were prepared by interfacial polycondensation. The procedure was optimized to enable the self-assembly, aggregation, and diffusion of supramolecular nanoaggregates within a conventional polyamide matrix. The HNDPA loading solution was prepared by dissolving 1.1 wt % of the HNDPA monomer in an ethanol–water mixture (EtOH:H_2_O, 70:30 v/v). As the HNDPA monomer self-assembles in an aqueous phase, the hydrogen-bond network is promoted by the solvent/water system.^42^ A commercial polysulfone membrane was loaded with the HNDPA solution and left to stand for approximately 60 s. Subsequently, the pores of the support layer were soaked with an aqueous solution of *m*-phenylenediamine (2 wt %) for 120 s. Excess solution was removed from the membrane surface using compressed air. The amine-loaded membrane was then exposed to an immiscible organic solution containing 0.1 wt % TMC for 60 s to initiate interfacial polymerization and form the nanocomposite active layer. A posttreatment involved the immersion in deionized (DI) water at 95 °C for 120 s, followed by rinsing with a 200 ppm of NaOCl aqueous solution for 120 s, soaking in a 1000 ppm of Na_2_S_2_O_5_ solution for 30 s, and a final rinse in DI water at 95 °C for 120 s. The membranes were stored in DI water at 4 °C until use.

### Atomic Force Microscopy (AFM)

For the AFM analysis, the samples were glued onto a glass microscope slide with the use of adhesive tape, ensuring that the sample lies flat and its back surface is in contact with the glass. The hydrated sample surface was covered with a few droplets of water on the surface, 30 min prior to the measurements and during the measurements. The analysis was done using Dimension Icon AFM (Bruker Corporation, MA, USA), a Scanasyst-Air AFM probe to scan the dry sample, and a Scanasyst-Fluid+ to scan the hydrated sample (Bruker Corporation, MA, USA). AFM data were analyzed using Gwyddion 2.68 software.

### Scanning Electron Microscopy (SEM)

For SEM analysis, dried membrane samples were mounted on aluminum SEM stubs with the use of PELCO double-sided carbon tape (Ted Pella Inc., CA, USA). To prepare cross sections of the membranes, samples were soaked in isopropyl alcohol for 1 min and subsequently immersed in liquid nitrogen and fractured, following a removal of delaminated nonwoven backing before mounting onto the carbon tape. The samples were stored in a drybox and coated by a turbomolecular pumped coater Q150T (Quorum Technologies, UK) with a 6 nm layer of iridium prior to SEM analysis. The SEM analysis was done at Magellan High-Resolution SEM (FEI Company, OR, USA), with an accelerating voltage of 5 kV, a current of 50 pA, and a working distance of 4 mm.

### Sample Preparation for PXCT

To prepare the TFC membranes for the PXCT experiments, the polyester nonwoven backing was separated from the polysulfone porous support, in a similar manner as described for the TEM sample preparation. The membrane (polyamide layer on polysulfone support) was placed on adhesive tape mounted on a laser microdissection frame slide (Leica Microsystems, Germany). The mounted membrane was cut using a laser microdissection system LMD7 (Leica Microsystems, Germany) into blocks of 15 μm × 15 μm, sectioned across the whole porous polysulfone support. The laser-cut blocks were collected on a microscope glass slide and carefully transferred under an Olympus SZX stereo microscope (Olympus, Japan) onto a 100 nm thick silicon nitride membrane window (2 mm membrane and 5 mm frame) (Silson Ltd., UK). To study the hydrated sample, a frame was placed around the membrane on the silicon nitride window, and water was deposited on the membrane, filling the space limited by the frame. A second silicon nitride membrane was placed over the membrane and frame as in Figure 1. To avoid evaporation during the measurements, the silicon nitride windows were glued by using Norland Optical UV curing Adhesive 81 (Norland Products, Inc., NJ, USA). The sample was hydrated for 1 h prior to PXCT measurements.

### Resolution Calculation Using Line Scans and Fourier Shell Correlation

The resolution of the ptycho-tomography images was estimated by using a line profile method in *XY* and *YZ* planes based on edge sharpness across selected interfaces between the active layer and the background in the reconstructed, unsegmented images. This approach involves extracting gray-value line profiles through high-contrast features in the dataset and computing the derivative of the intensity profile to highlight transition regions. A Gaussian function is then fitted to this derivative, and the full width at half-maximum (fwhm) of the fitted Gaussian provides a quantitative measure of the local spatial resolution. This method aligns with the 25–75% edge response criterion, where resolution is defined by the width over which the intensity increases from 25 to 75% of the gray value across an edge.^55–57^ Line profiles were applied at different image slices from each sample, in the sample and air interface for dry and sample and water interface for hydrated sample, resulting in calculated spatial resolutions of 70 (±5) and 70 (±3) nm for the dry and 69 (±5) and 69 (±2) nm for the hydrated sample, in the *XY* and *YZ* planes, respectively (Figure S11). The Fourier shell correlation analysis was performed using PtychoShelves using the 1/2 bit criterion and resulted in resolutions of 111 nm for dry and 112 nm for hydrated sample. The Fourier shell correlation curves are presented in Figure S12.

### Segmentation, 3D Visualization, Volume, Surface Area, and Roughness Calculations

The reconstructed tomographic image stacks were processed by using Avizo 3D 2022.2 (Thermo Fisher Scientific, MA, USA). From the generated 3D volume of the whole scanned region of the membrane, regions of interest were selected. The regions of interest correspond to 6 × 6 μm surface areas of the polyamide active layer of the membrane, where the artifacts resulting from the missing wedge were the least pronounced. The regions of interest images were segmented manually slice-by-slice, using a Wacom Cintiq Pro tablet and pen (Wacom Co., Ltd., Japan). For the 3D visualization, volume rendering with cubic interpolation was used. Quantitative data regarding roughness and volumes were calculated based on 31 nm × 31 nm × 31 nm voxel datasets.

Avizo *modules* were used to quantify volumes, surface areas, and heights (used for roughness) of the segmentation data. Volume was measured using *Label Analysis* module with *Volume3D* measurement applied to each target segmentation’s binary label data. To quantify the surface area, a triangular mesh representation of each segmentation is first computed using the *Generate Surface* module. Then, a *Surface Area Volume* module is applied to the resulting mesh to measure the final surface area. Since Avizo does not have a built-in roughness measurement module, the height map, i.e., vertical heights of each *x* and *y* coordinate in the segmentation, was first extracted and then fed to an external tool to compute the roughness. To do this, each vertical segment (unique *x*,*y* coordinate) was assigned a unique label index using an *Arithmetic* module followed by a *Label Analysis* that measures *BoundingBoxDz*, which results in the desired height information that is eventually used for the roughness computations.

The height data were used to compute roughness parameters, such as arithmetic average roughness (*R*_a_), provided in eq 1:

$$R_{a}=\frac{1}{n}\sum_{i=1}^{n}|y_{i}|$$ 1

where *n* is the total number of collected points and *y* is the profile height deviation from the mean line.

Root-mean-square roughness (*R*_q_) was calculated with eq 2:

$$R_{q}=\sqrt{\frac{1}{n}\sum_{i=1}^{n}y_{i}^{2}}$$ 2

Skewness (*R*_sk_) was calculated with eq 3:

$$R_{\mathrm{sk}}=\frac{1}{n\cdot R_{q}^{3}}\sum_{i=1}^{n}y_{i}^{3}$$ 3

Kurtosis (*R*_ku_) was calculated with eq 4:

$$R_{\mathrm{ku}}=\frac{1}{n\cdot R_{q}^{4}}\sum_{i=1}^{n}y_{i}^{4}$$ 4

## Supplementary Material



















## Notes

Angular coverage and missing wedges of PXCT analysis (Figure S1), aligned sinograms after the tomographic consistency alignment, obtained from the PtychoShelves tomographic reconstruction script (Figure S2), presegmentation membrane images obtained by PXCT (Figure S3), original images of dry and wet membranes (Figure S4), exemplary orthogonal slices of the reconstructed PXCT data (Figure S5), PXCT visualizations (Figures S6–S8), features protrude from the surface (Figure S9), scanning electron microscopy (Figure S10), exemplary line profile calculations (Figure S11), Fourier shell correlation curves (Figure S12), volume comparison of selected polyamide surface features (Table S1), and summary of the data collected with AFM and PXCT (Table S2) (PDF)
(Video S1) Presegmentation reconstruction of dry membrane projections (low magnification, large volume observation), before alignment (MP4)
(Video S2) Presegmentation reconstruction of dry membrane projections (low magnification, large volume observation), after alignment (MP4)
(Video S3) Presegmentation reconstruction of hydrated membrane projections (low magnification, large volume observation), before alignment (MP4)
(Video S4) Presegmentation reconstruction of hydrated membrane projections (low magnification, large volume observation), after alignment (MP4)
(Video S5) Presegmentation reconstruction of dry membrane projections (high magnification of a selected volume showing the polyamide layer and part of the porous support) (MOV)
(Video S6) Presegmentation reconstruction of hydrated membrane projections (high magnification of a selected volume showing the polyamide layer and part of the porous support) (MOV)
(Video S7) 3D visualization of the dry polyamide layer after segmentation (MOV)
(Video S8) 3D visualization of the hydrated polyamide layer after segmentation (MOV)
