# Supplementary material for: In Situ Ptychographic X‑ray Computed Tomography of Fully Hydrated Polyamide Membranes
Source: Langmuir. 2026 Jan 30;42(5):3828–38. doi: 10.1021/acs.langmuir.5c04933 (PMC12895506; doi:10.1021/acs.langmuir.5c04933)
Supplement: Supplementary file 1 [file la5c04933_si_001.pdf]

Supporting information

In situ ptychographic x-ray computed tomography of fully hydrated polyamide membranes

Radosław Górecki [1], Ronell Sicat [2], Carla Cristina Polo [3], Tiago Araujo Kalile [3],
Maria Di Vincenzo [1], Florian Meneau [3, 4], Suzana P. Nunes [1, 5, 6*]

[1] Environmental Science and Engineering Program, Biological and Environmental Science and Engineering, King Abdullah University of Science and Technology (KAUST), 23955-6900, Thuwal, Saudi Arabia

[2] Visualization Core Labs, King Abdullah University of Science and Technology (KAUST), 23955-6900, Thuwal, Saudi Arabia

[3] Brazilian Synchrotron Light Laboratory (LNLS), Brazilian Center for Research in Energy and Materials (CNPEM), 13083-970, Campinas, SP, Brazil

[4] Institute of Chemistry, University of Campinas (UNICAMP), 13083-970, Campinas, SP, Brazil

[5] Chemistry Program, Physical Science and Engineering, King Abdullah University of Science and Technology (KAUST), 23955-6900, Thuwal, Saudi Arabia

[6] Chemical Engineering Program, Physical Science and Engineering, King Abdullah University of Science and Technology (KAUST), 23955-6900, Thuwal, Saudi Arabia

*Corresponding author: suzana.nunes@kaust.edu.sa

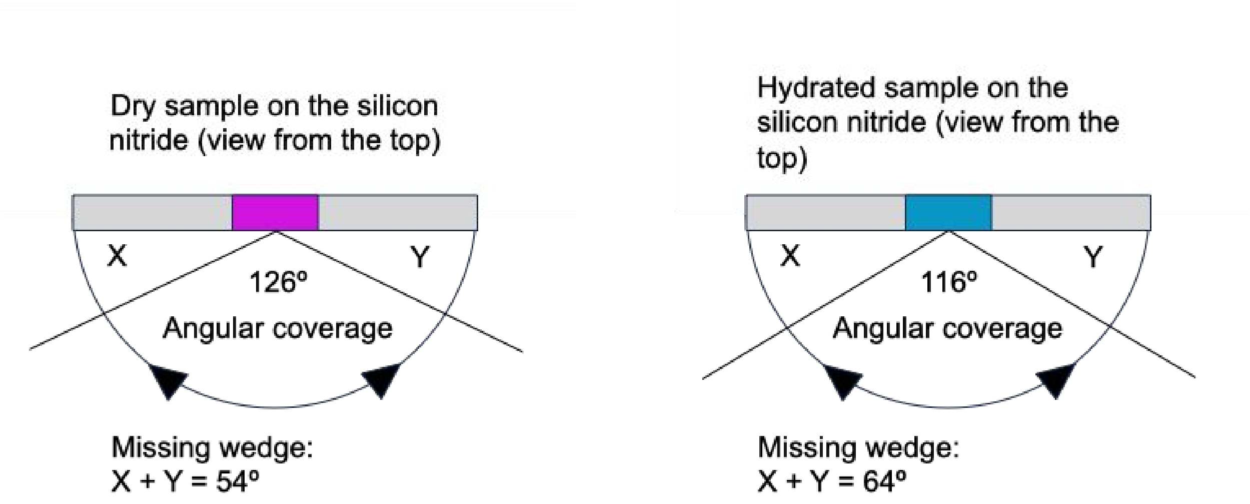

Figure S1. Angular coverage and missing wedges of PXCT analysis of dry and hydrated membranes.

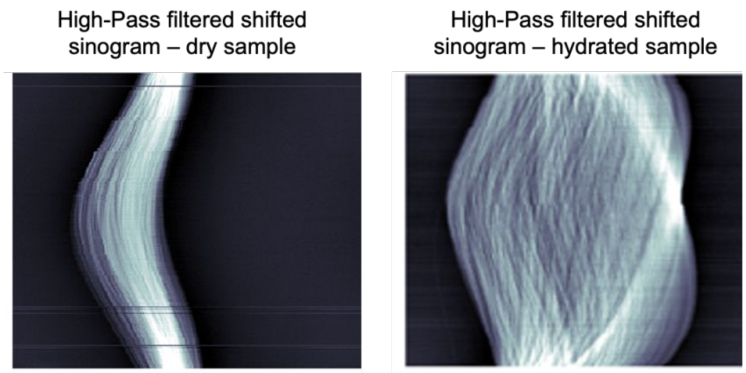

Figure S2. aligned sinograms after the tomographic consistency alignment, obtained from PtychoShelves tomographic reconstruction script for dry and hydrated samples.

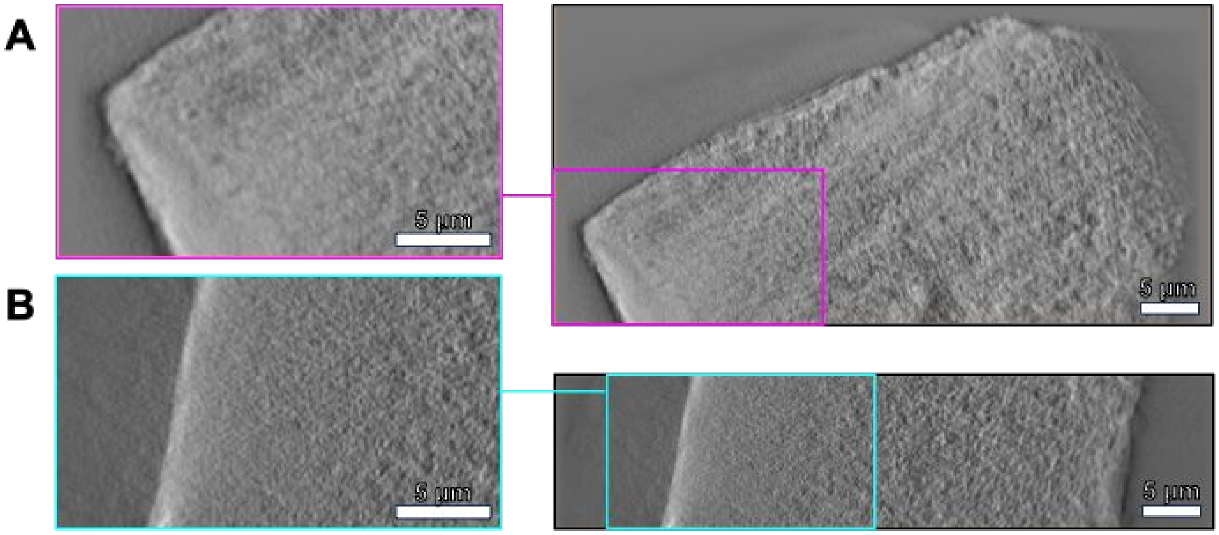

Figure S3. Pre-segmentation membrane images obtained by PXCT, showing the top polyamide layer and the polysulfone porous support for (A) dry membrane, and (B) hydrated membrane.

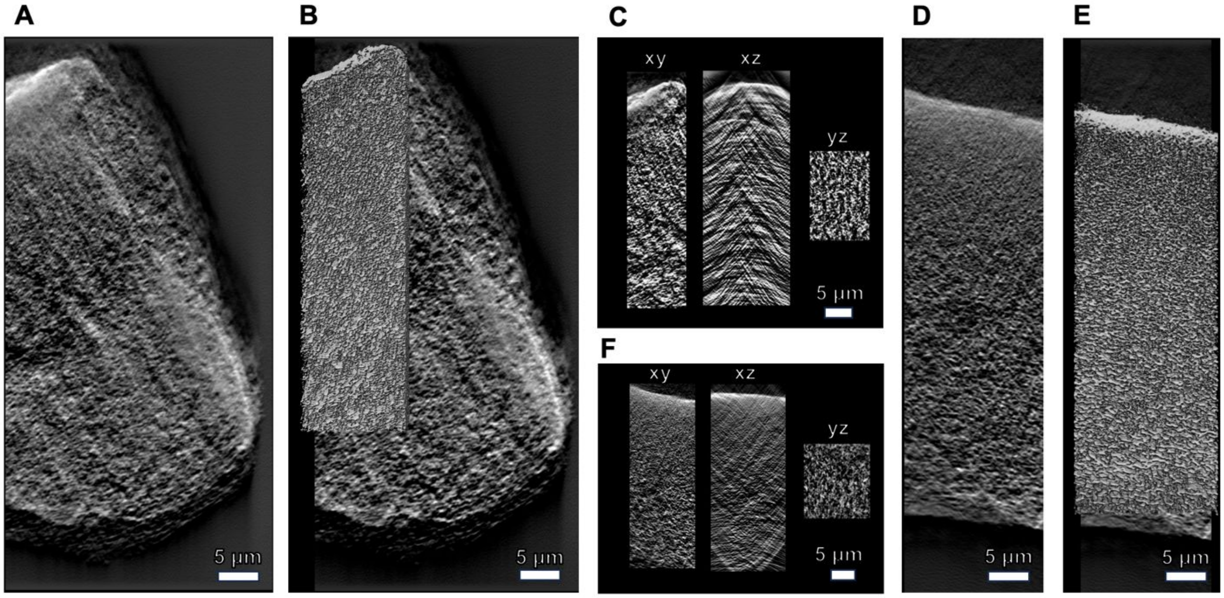

Figure S4. Original images of (A-C) dry and (D-F) wet membranes. (A) and (D) are pre-segmentation membrane images obtained by PXCT, produced by PtychoShelves software, showing the top polyamide layer and the polysulfone porous support. (B) and (E) are the regions of the reconstruction selected for representation in 3D. (C) and (F) present unmodified orthogonal slices through the selected region in xy, xz, and yz directions.

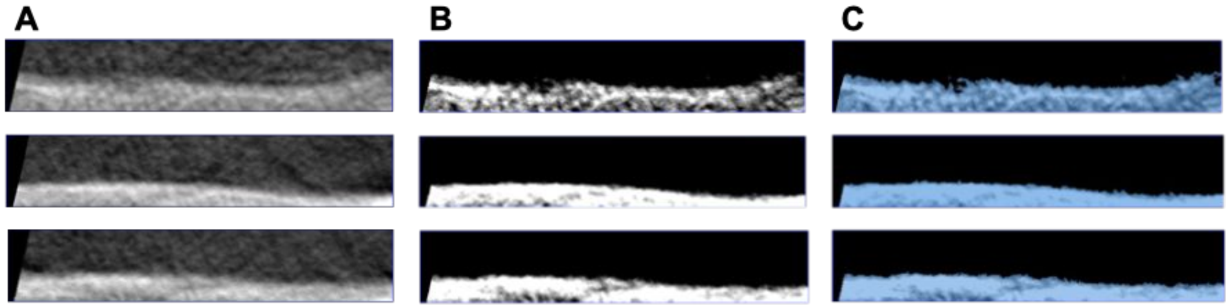

Figure S5. Exemplary orthogonal slices of the reconstructed PXCT data, (A) before removal of the background signal (B) after removal of the background signal, and (C) after manual segmentation for the hydrated membrane sample. The area marked in blue is a hand-segmented data taken into account as a material for 3D visualizations.

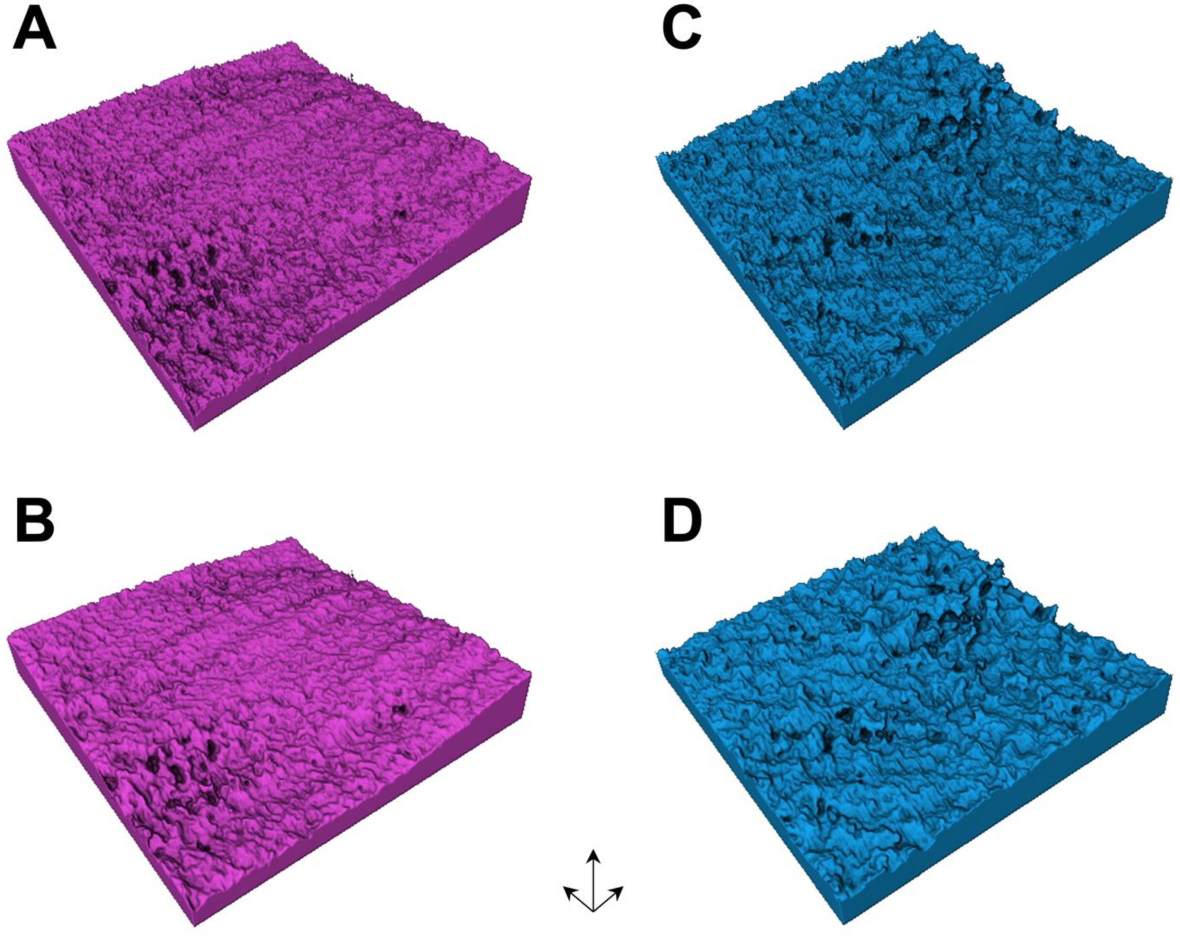

Figure S6. PXCT Visualizations of (A) dry sample before cubic interpolation and (B) dry sample after cubic interpolation. PXCT Visualizations of (C) hydrated sample before cubic interpolation and (D) hydrated sample after cubic interpolation. Scale bars are 500 nm in all directions.

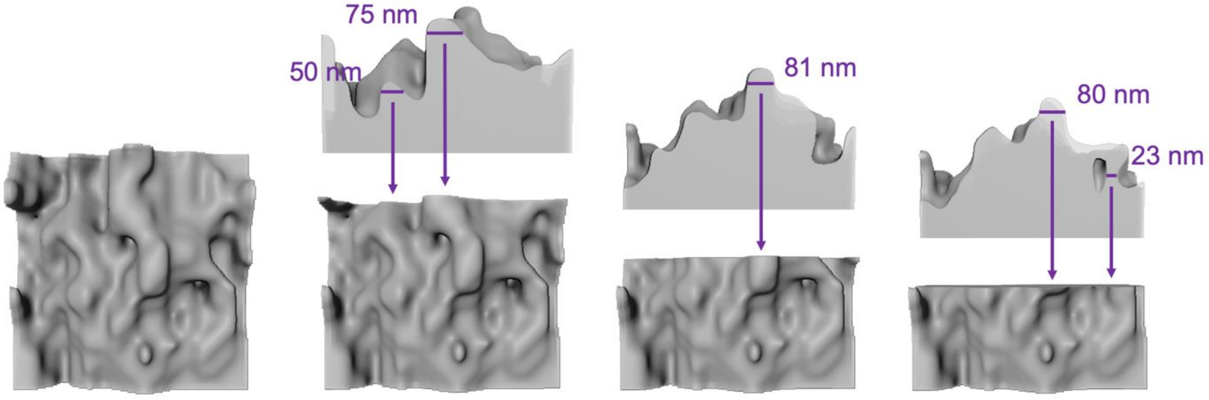

Figure S7. PXCT visualization orthogonal slice section through the bowl-like nodules and their ridges in dry polyamide.

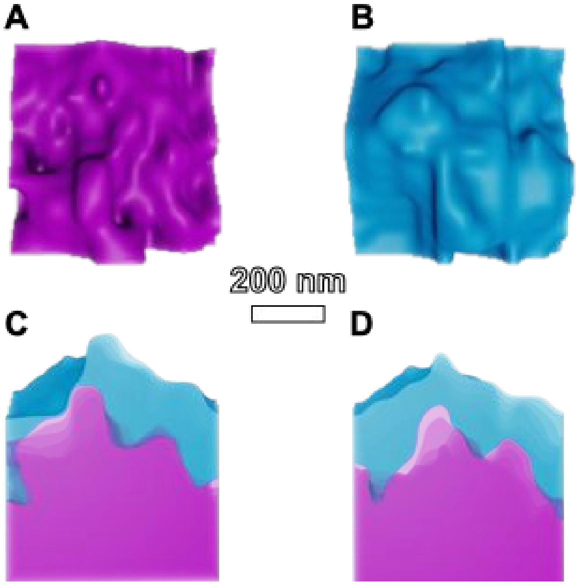

Figure S8. PXCT visualization comparison of the bowl-like nodules in (A) dry and (B) hydrated states, (C) (D) marking the dry image contribution as magenta, and the remaining part of the hydrated membrane image as blue.

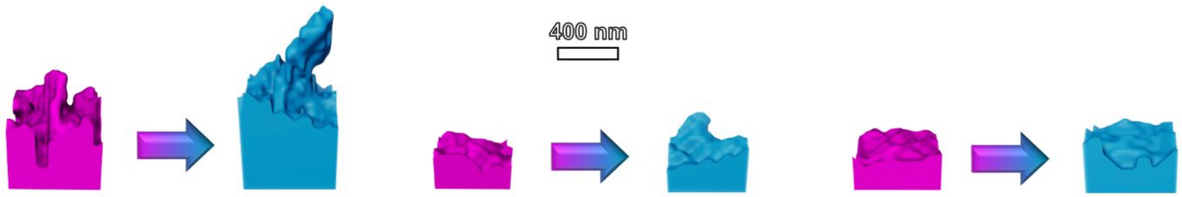

Figure S9. Features protrude from the surface observed in the dry state (magenta) and in water (blue).

Table S1. Volume comparison of selected polyamide surface features in dry and hydrated states. The table summarizes the volumes of individual nodular features with similar morphology before and after hydration (from Figure 4), along with the corresponding percentage change. All volumes were extracted from the reconstructed PXCT datasets.

Feature	Dry Volume ($10^7 nm^3$)	Hydrated Volume (10^7 nm^3)	Change in Volume
I	8.4	11.7	38%
II	3.9	5.8	49%
III	1.2	1.6	39%
IV	5.7	7.4	30%
V	2.9	4.4	51%
VI	2.7	3.5	29%

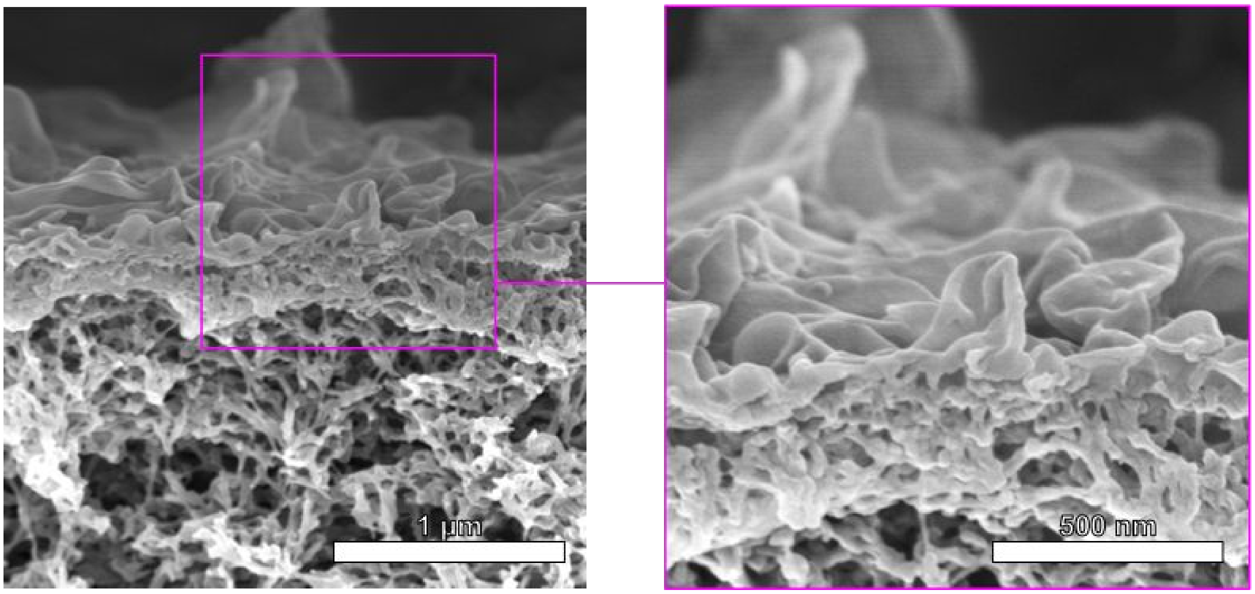

Figure S10. Scanning electron microscopy of the cross-section of thin film composite polyamide membrane with 2-hydroxy-N-(diphenylmethyl)acetamide artificial water channels.

Table S2. Summary of the data collected with atomic force microscopy (AFM) and ptychographic x-ray computed tomography (PXCT) including pixel size, surface area, arithmetic average roughness R_a, root mean square roughness R_q, skewness R_{sk} and kurtosis R_{ku}.

	**Dry Sample**		**Hydrated Sample**	
	**AFM**	**PXCT**	**AFM**	**PXCT**
**Pixel Size**	8 nm	31 nm	8 nm	31 nm
**Surface Area**	8.8 µm²	8.5 µm²	9.2 µm²	13.1 µm²
**Arithmetic Average Roughness R_a**	93 nm	50 nm	120 nm	114 nm
**Root Mean Square Roughness R_q**	111 nm	62 nm	150 nm	135 nm
**Skewness R_{sk}**	0.16	-0.09	0.23	0.02
**Kurtosis R_{ku}**	2.81	3.31	3.8	2.57

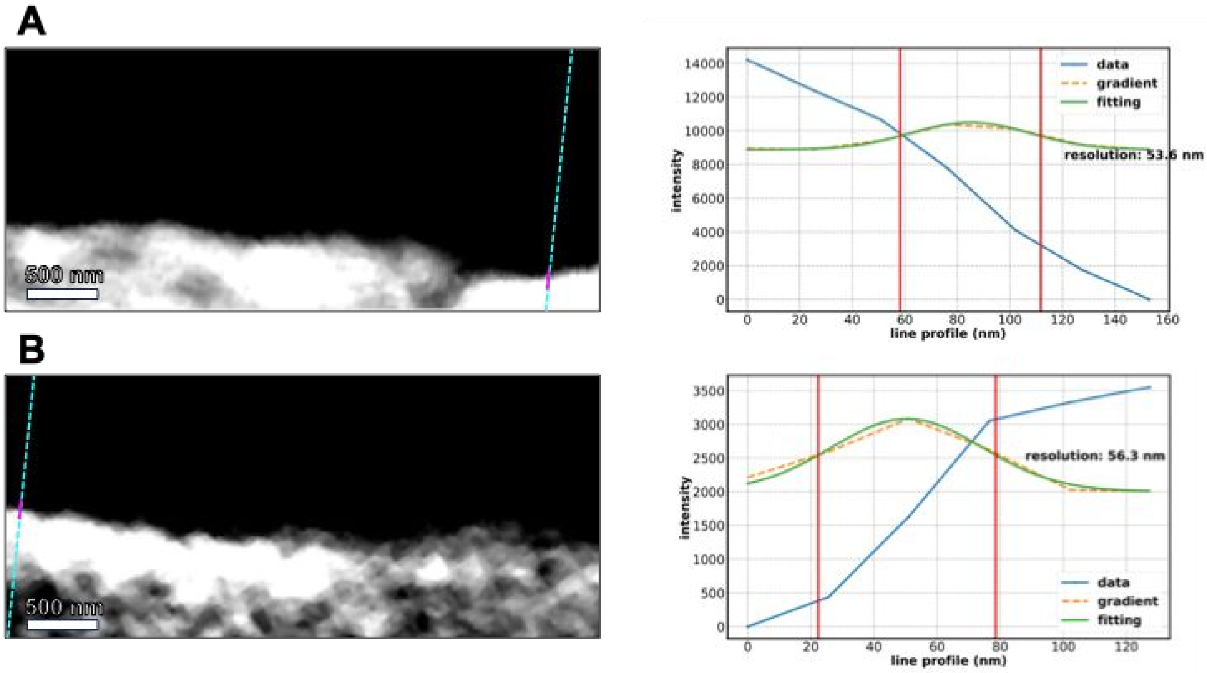

Figure S11. Exemplary line profile calculations for (A) dry sample, at the sample and air interface, and for (B) hydrated sample, at the sample and water interface.

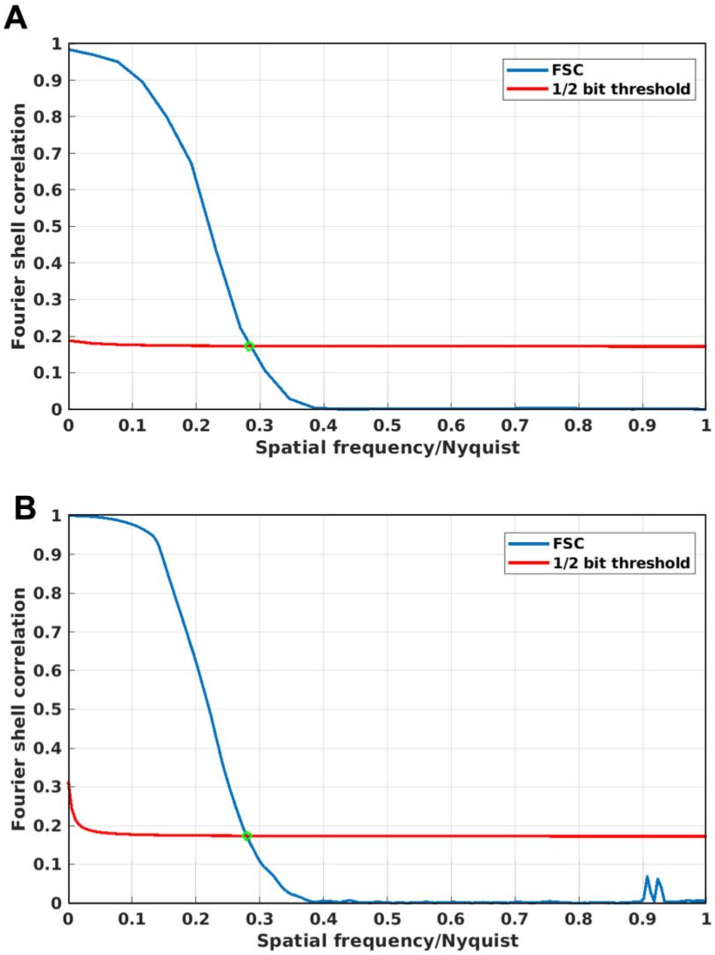

Figure S12. Fourier shell correlation curves obtained from PtychoShelves for (A) dry sample, at the sample and air interface, and for (B) hydrated sample, at the sample and water interface.
